# Bergamot Polyphenolic Extract Combined with Albedo and Pulp Fibres Counteracts Changes in Gut Microbiota Associated with High-Fat Diet: Implications for Lipoprotein Size Re-Arrangement

**DOI:** 10.3390/ijms241612967

**Published:** 2023-08-19

**Authors:** Rocco Mollace, Roberta Macrì, Martina Nicita, Vincenzo Musolino, Micaela Gliozzi, Cristina Carresi, Irene Bava, Jessica Maiuolo, Annamaria Tavernese, Antonio Cardamone, Luigi Tucci, Giuseppe Trunfio, Elzbieta Janda, Ernesto Palma, Carolina Muscoli, Francesco Barillà, Massimo Federici, Federica Scarano, Vincenzo Mollace

**Affiliations:** 1 Pharmacology Laboratory, Institute of Research for Food Safety and Health IRC-FSH, Department of Health Sciences, University Magna Graecia of Catanzaro, 88100 Catanzaro, Italy; rocco.mollace@gmail.com (R.M.); n.marty96@hotmail.com (M.N.); micaela.gliozzi@gmail.com (M.G.); irenebava@libero.it (I.B.); an.tavernese@gmail.com (A.T.); tony.c@outlook.it (A.C.); l.tucci@head-sa.com (L.T.); g.trunfio@head-sa.com (G.T.); janda@unicz.it (E.J.); muscoli@unicz.it (C.M.); federicascar87@gmail.com (F.S.); 2 Department of Systems Medicine, University of Rome Tor Vergata, 00133 Roma, Italy; francesco.barilla@uniroma2.it (F.B.); federicm@uniroma2.it (M.F.); 3 Pharmaceutical Biology Laboratory, Institute of Research for Food Safety and Health IRC-FSH, Department of Health Sciences, University Magna Graecia of Catanzaro, 88100 Catanzaro, Italy; v.musolino@unicz.it (V.M.); maiuolo@unicz.it (J.M.); 4 Veterinary Pharmacology Laboratory, Institute of Research for Food Safety and Health IRC-FSH, Department of Health Sciences, University Magna Graecia of Catanzaro, 88100 Catanzaro, Italy; carresi@unicz.it (C.C.); palma@unicz.it (E.P.); 5 Renato Dulbecco Institute, Lamezia Terme, 88046 Catanzaro, Italy

**Keywords:** gut microbiota, atherosclerotic vascular disease, high-fat diet, lipoprotein assembly, lipid metabolism, bergamot extract, polyphenols, prebiotics

## Abstract

Evidence exists that the gut microbiota contributes to the alterations of lipid metabolism associated with high-fat diet (HFD). Moreover, the gut microbiota has been found to modulate the metabolism and absorption of dietary lipids, thereby affecting the formation of lipoproteins occurring at the intestinal level as well as systemically, though the pathophysiological implication of altered microbiota composition in HFD and its role in the development of atherosclerotic vascular disease (ATVD) remain to be better clarified. Recently, evidence has been collected indicating that supplementation with natural polyphenols and fibres accounts for an improvement of HFD-associated intestinal dysbiosis, thereby leading to improved lipidaemic profile. This study aimed to investigate the protective effect of a bergamot polyphenolic extract (BPE) containing 48% polyphenols enriched with albedo and pulp-derived micronized fibres (BMF) in the gut microbiota of HFD-induced dyslipidaemia. In particular, rats that received an HFD over a period of four consecutive weeks showed a significant increase in plasma cholesterol, triglycerides and plasma glucose compared to a normal-fat diet (NFD) group. This effect was accompanied by body weight increase and alteration of lipoprotein size and concentration, followed by high levels of MDA, a biomarker of lipid peroxidation. Treatment with a combination of BPE plus BMF (50/50%) resulted in a significant reduction in alterations of the metabolic parameters found in HFD-fed rats, an effect associated with increased size of lipoproteins. Furthermore, the effect of BPE plus BMF treatment on metabolic balance and lipoprotein size re-arrangement was associated with reduced gut-derived lipopolysaccharide (LPS) levels, an effect subsequent to improved gut microbiota as expressed by modulation of the Gram-negative bacteria Proteobacteria, as well as Firmicutes and Bacteroidetes. This study suggests that nutraceutical supplementation of HFD-fed rats with BPE and BMP or with their combination product leads to restored gut microbiota, an effect associated with lipoprotein size re-arrangement and better lipidaemic and metabolic profiles.

## 1. Introduction

Evidence has been accumulated showing that cardiovascular disease states, including myocardial infarction, hypertension, stroke and peripheral vascular diseases, may be affected by alterations of gut microbiota [1,2]. The pathophysiological basis of these correlations is still unknown. However, data have been provided that multiple bio-molecular pathways appear to be involved in changes occurring in gut microbiota which increase cardiometabolic risk, mostly related to infectious conditions, modifications of host bile acid and changes in lipidaemic profile [3,4,5]. These events are supposed to contribute to an enhanced passage of endotoxins or toxic metabolites from the intestine to the bloodstream, an effect potentially associated with pro-atherogenic effects, primarily through the acceleration of pre-existing atherosclerotic vascular lesions (e.g., inflammatory-related atherosclerotic plaque destabilization) [6,7]. Consistent data show that endotoxins such as *E. coli* lipopolysaccharide (LPS) deriving from gut Gram-negative bacteria represent potential enhancers for the development of atherosclerotic plaque, possibly via activation of smooth muscle cell proliferation [8,9,10,11]. In particular, LPS was found to activate cytokine release, an effect associated with the activation of Toll-like receptor (TLR) signalling, and promote LDL oxidation, thereby reducing constitutive NO release and promoting lipid peroxidation via harmful peroxynitrite generation [12]. Thus, the increase in circulating endotoxin levels as a consequence of gut microbiota disturbances could play a role in atherosclerosis development. On the other hand, bacterial metabolites, such as trimethylamine (TMA), are transformed in the liver into trimethylamine N-oxide (TMAO), which at high levels is associated with the development of non-alcoholic fatty liver disease (NAFLD) [13,14], an effect which is accompanied by altered metabolic balance, re-arrangement of lipoprotein composition and, finally, enhanced atherosclerosis development [15,16]. These data are partially confirmed by clinical observational studies in which modifications of gut microbiota seem to clearly correlate with increased cardiometabolic risk, though specific changes have not been identified yet and results are often not corroborated between studies [17,18].

Natural products claimed to produce potential benefits in modulating gut microbiota disorders have been correlated with an improvement of cardiometabolic profile in both animal models of cardiovascular disease and in patients [19,20,21]. Evidence has been shown that the use of prebiotics alone or in combination with natural antioxidants stimulates the growth of beneficial gut bacteria, leading to health benefit [22,23].

In particular, both pre-clinical and clinical studies have highlighted the possible beneficial responses to counteract the development of cardiovascular disease produced by several prebiotics, including oligosaccharides, inulin and pectins as the ones found in natural fibres deriving from plant derivatives [24]. These responses depend on an increased production of short-chain fatty acids (SCFAs) accounted for the growth of beneficial bacteria genera, such as *Lactobacillus* and *Bifidobacterium* [25]. In addition, SCFA increase leads to an antagonistic effect on the growth of pathogenic species [26,27] and to better intestinal epithelial cell layer protection [28]. Finally, SCFAs have been found to inhibit histone deacetylase, thereby contributing to resolving gut inflammatory conditions [29,30], leading also to better systemic metabolic balance via activating glucagon-like peptide-1 (GLP-1) production [31].

Besides these effects, the contribution of prebiotics to gut-microbiota-related disorders remains to be better clarified.

Alongside prebiotics, diet supplementation with natural plant-derived compounds, such as flavonoids, a family of active ingredients well represented in *Citrus* derivatives, berries, red wine, apples and olive oil, have widely been correlated with improvement of gut microbial dysbiosis [32,33]. The contribution of such natural antioxidants to prevent cardiometabolic disorders accompanying gut microbiota alterations may depend on various effects mostly related to their properties in scavenging the overproduction of free radical species (ROS) [34,35]. In particular, data exist that flavonoids such as naringin and hesperidin possess an inhibitory effect on TMA-lyase, thereby leading to reduced TMAO concentration [36,37]. Therefore, the combination of flavonoids and fibres with prebiotic properties may represent a valid solution for approaching cardiometabolic disorders thanks to their potential synergistic response under conditions such as high fat diet (HFD), in which an alteration of lipidaemic profile is associated with altered gut microbiota [38,39].

In recent years, evidence has been collected that *Citrus bergamia* Risso & Poiteau (bergamot), a *Citrus* species rich in flavonoids, may represent a potential source of natural antioxidants able to counteract HFD-induced alteration of lipidaemic profile both in animal models of hyperlipidaemia and in patients [40,41]. Moreover, a polyphenolic-rich extract derived from bergamot juice has been found to improve lipoprotein profiles in patients with liver steatosis alongside having a beneficial effect on liver function [42]. Finally, bergamot fibres alone or in combination with natural polyphenolic extracts were found to be able to antagonize fat accumulation and insulinaemic response both in animals and obese patients, suggesting that dietary supplementation with such products may have a beneficial response in metabolic regulation under HFD conditions [43,44].

Here, we studied the effect of a bergamot-derived polyphenolic-rich extract (BPE), combined with bergamot fibres (BMP) prebiotic-rich in pectin and oligosaccharides derived from bergamot albedo and pulp fibres, on the gut microbiota of rats fed a normal (NFD) and a high-fat diet (HFD). Data for this unique preparation on gut microbiota were correlated with lipidaemic profile, oxidative/inflammatory response and, finally, lipoprotein size and concentration.

## 2. Results

### 2.1. Effect of BPE, BMF and BPE + BMF on Lipidaemic Profiles in Rats Fed a NFD and a HFD

**Figure 1:**
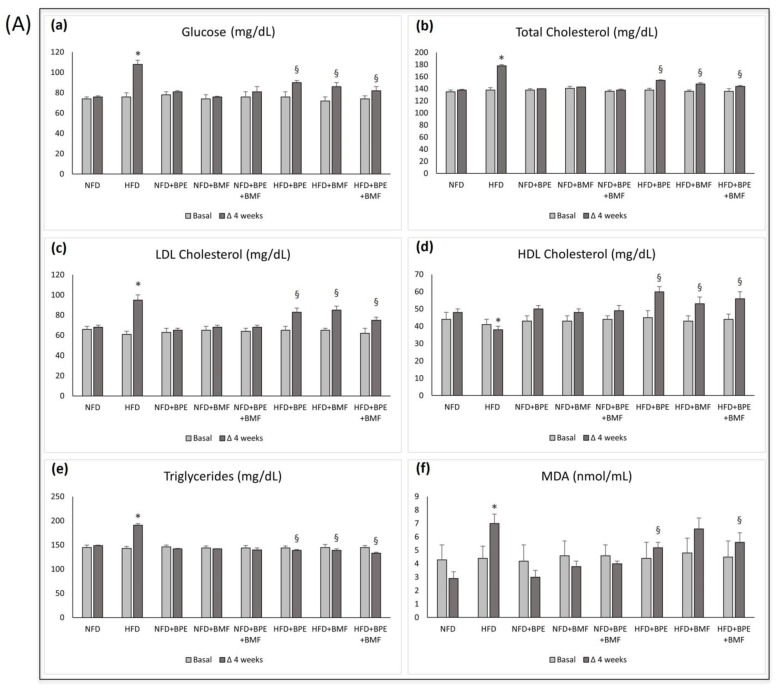

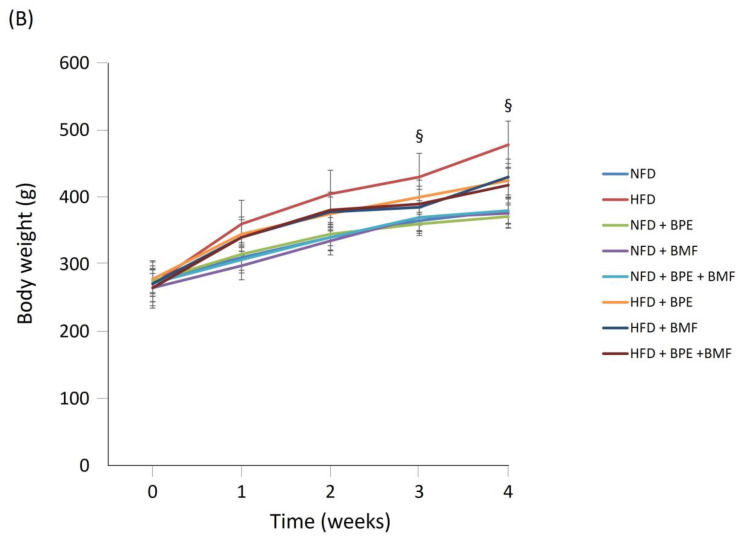
The effect of BPE, BMF or BPE + BMF (20 mg/Kg daily given orally over a period of 4 weeks on (**A**) plasma glucose (**a**), total cholesterol (**b**), LDL cholesterol (**c**), HDL cholesterol (**d**), triglycerides (**e**) and MDA (**f**) and on (**B**) body weight in NFD and HFD groups. Data are expressed as means ± SEs. *: *p* < 0.05 vs. NFD; §: *p* < 0.05 vs. HFD.

Supplementation of rats with an HFD was associated with metabolic changes and body weight alterations compared to rats fed a NFD. In particular, in rats fed a HFD over a period of four consecutive weeks, an increase in body weight, fasting plasma glucose, total cholesterol, LDL cholesterol, HDL cholesterol, triglycerides and MDA levels was found compared to rats receiving the standard diet (NFD) (Figure 1A,B). This effect was counteracted by supplementation of rats with BPE, BMF and BPE + BMF, as shown in Figure 1A,B. Indeed, in animals fed a HFD and supplemented with 20 mg/kg of BPE, BMF and BPE + BMF in a single daily administration via gastric gavage, a significant reduction in all parameters was found compared to the ones altered by HFD alone. No effect was found in rats receiving a standard diet (NFD) compared to groups supplemented with BPE, BMF and BPE + BMF at 20 mg/kg (Figure 1A,B). Moreover, all parameters at baseline were comparable in all eight groups of rats used throughout the study.

**Figure 2:**
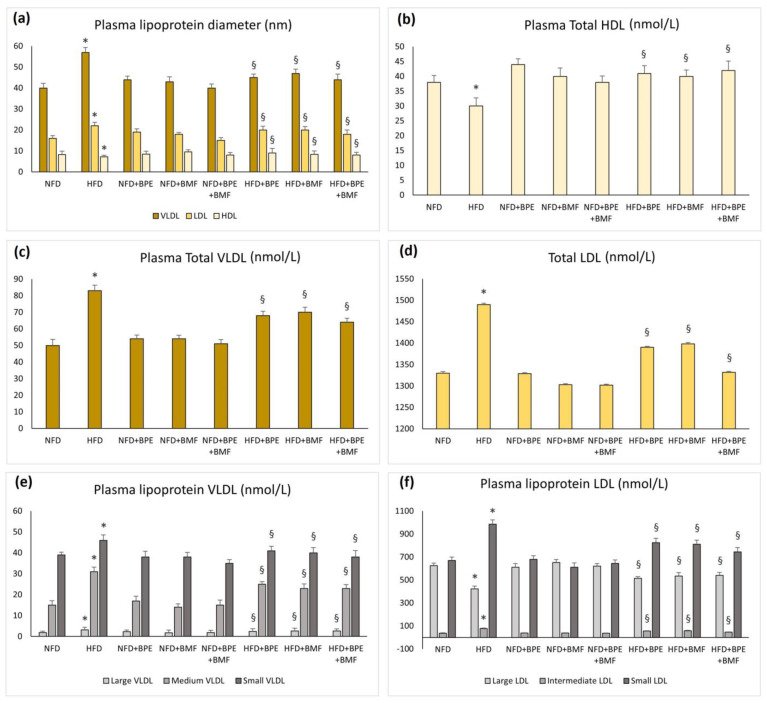
The effect of BPF, BMF or BPF + BMF (20 mg/Kg daily given orally over a period of 4 weeks) on lipoprotein diameter (**a**) and concentration (nmol/L) in NFD and HFD groups. Plasma total HDL (**b**); plasma total VLDL (**c**); plasma total LDL (**d**); plasma lipoprotein VLDL (large, medium and small) (**e**); plasma lipoprotein LDL (large, intermediate and small) (**f**). Data are expressed as means ± SEs. *: *p* < 0.05 vs. NFD; §: *p* < 0.05 vs. HFD.

The effect of treatment with bergamot extracts was also found to be able to modify HFD-induced changes of lipoprotein profile compared to the NFD. Indeed, the HFD compared to the NFD produced a substantial re-arrangement of plasma levels of lipoprotein particles in rats as evaluated after 4 weeks compared to basal levels (Figure 2).

This effect was counteracted by treatment for 4 weeks with 20 mg/Kg of BPE, BMF or BPE + BMF (Figure 2). In particular, BPE + BMF was found to be able to decrease the mean concentration of IDL particles by 40.26%, to increase large LDLs by 27.6% and to decrease small LDLs by 24.4%. On the other hand, treatment with BPE + BMF led to a 40% increase in total HDL particles, mainly due to the increase in large HDL particles.

### 2.2. Effect of BPE, BMF and BPE + BMF on Gut Microbiota in Rats NFD and a HFD

**Figure 3:**
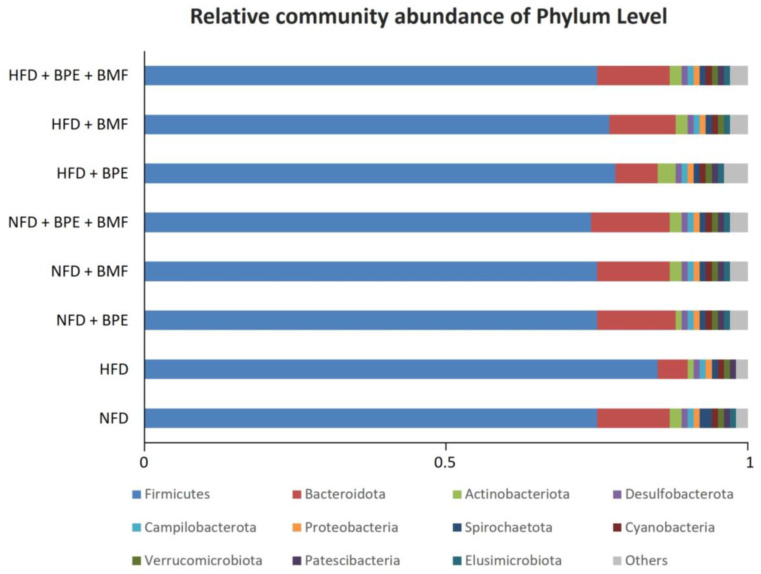
The modulatory effect of BPE, BMF or BPE + BMF on the gut microbiota composition (determined by sequencing the V3–V4 region of the 16S rRNA gene using the MiSeq Illumina system) expressed as relative community abundance at the phylum level. Among the identifiable bacterial phyla with ≥1% abundance in all samples, Firmicutes was the most abundant, followed by Bacteroidota and Actinobacteroidota.

The studies on gut microbiota were carried out in order to verify the microbial composition and abundance at the phylum level in both NFD and HFD rats either untreated or treated with BPE, BMF and BPE + BMF (Figure 3).

**Figure 4:**
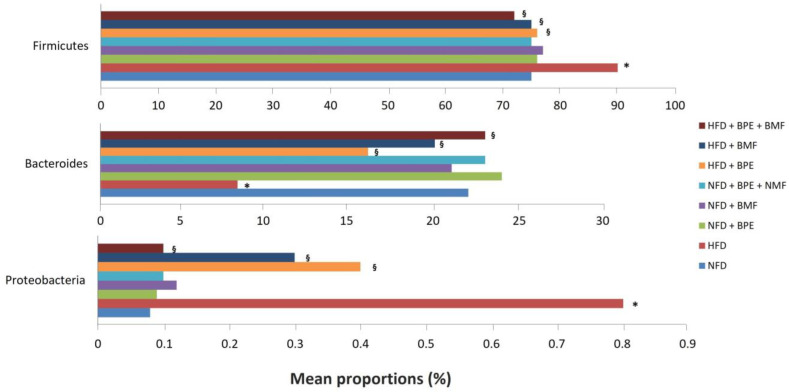
The effect of BPE, BMF or BPE + BMF on the relative abundances of Firmicutes, Bacteroidetes and Proteobacteria in NFD and HFD groups. Data are expressed as means ± SEs (n = 6). *: *p* < 0.05 vs. the NFD group; §: *p* < 0.05 vs. the HFD group.

In particular, under basal conditions, 11 phyla were found in eight groups, showing that Firmicutes and Bacteroidetes represented the largest proportions among detectable bacteria. However, in animals fed a HFD, compared to the NFD group, consistent changes were found in our study. Indeed, increased proportions of Firmicutes and Protobacteria were found as a consequence of HFD supplementation compared to the NFD and, in contrast, a significant decrease in Bacteroidetes was observed in the HFD group. This effect was counteracted by treating rats with BPE, BMF and BMF + BPE (Figure 4).

Indeed, all bergamot derivatives almost completely attenuated changes produced by the HFD compared to the NFD, showing that the combination of polyphenols and bergamot fibres may contribute to restore the normal composition of gut microbiota.

### 2.3. Effect of BPE, BMF and BPE + BMF on Plasma LPS Levels in Rats Fed a NFD and a HFD

**Figure 5:**
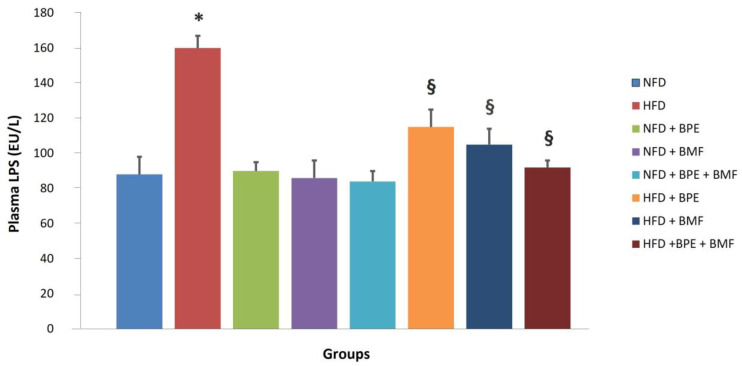
The effect of BPF, BMF or BPF + BMF (20 mg/Kg daily given orally over a period of 4 weeks) on plasma LPS (EU/L) concentration in NFD and HFD groups. Data are expressed as means ± SE. *: *p* < 0.05 HFD vs. NFD; §: *p* < 0.05 HFD.

Plasma LPS levels detected after four weeks were significantly higher in the HFD group compared to the NFD group (Figure 4). Treatment of rats with BPE, BMF or BPE + BMF resulted in significant lowering of plasma LPS levels compared to the HFD group. No changes of plasma LPS concentration were found in NFD-fed rats either untreated or treated with BPE, BMF or BPE + BMF (Figure 5).

## 3. Discussion

Our data show that a hyperlipidaemic diet (HFD) led to alterations of gut microbiota which resulted in a significant increase in the abundance of Firmicutes and Proteobacteria and a decreased abundance of Bacteroidetes compared to rats fed a NFD. This effect was associated with increased LPS levels in rats fed the HFD, thereby confirming that changes of gut microbiota subsequent to HFD lead to an inflammatory state combined with oxidative stress as detected by means of MDA measurements [45,46,47]. The changes of gut microbiota found after 4 weeks of HFD were associated with increased body weights and metabolic alterations represented by increased plasma glucose, cholesterol and triglycerides and changes of lipoprotein size and concentration compared with rats fed a normolipaemic diet (NFD). Moreover, oxidative stress biomarkers such as MDA were also found to be elevated in the blood of HFD rats, as previously shown by our and other groups [48,49,50].

These effects were counteracted by treating rats with BPE alone or in combination with BMF. In fact, in rats fed a HFD, where the diet was supplemented with BPE, BMP or a combination of both, the normal pattern of gut microbiota was restored, this effect being associated with reduction in body weight and in plasma levels of glucose, cholesterol and triglycerides. Interestingly, the improvement of lipidaemic profiles in rats receiving bergamot extracts alone or in combination with fibres was accompanied by an increased size of lipoproteins, mostly LDLs, which are known to play a key role in atherosclerosis development. On the other hand, the effect of natural antioxidants combined with BMF was associated with reduced LPS levels and with consistent reduction in MDA, thereby leading to an overall improvement of cardiometabolic risk profile in rats fed with HFD.

Our data are consistent with previous observations showing that the gut microbiota is a key player in modulating dietary lipid metabolism, affecting almost all the steps involved in the regulation of lipid digestion and absorption, being also involved in the generation of lipoproteins occurring at the intestinal level [15]. In particular, it has been shown that changes in gut microbiota composition, such as the one obtained by means of supplementation with Gram-negative bacteria, lead to obesity with increased LDL lipoproteins, an effect accompanied by elevation of plasma cholesterol and modulation of the lipid transfer protein system [51]. On the other hand, germ-free mice develop an obesity-resistant phenotype when fed a HFD, an effect which involves decreased fasting triglycerides and VLDL production when compared to conventionally reared mice [52].

Moreover, our data confirm previous evidence showing that HFD leads to modifications of gut microbiota [53]. On the other hand, our data confirm that restoring the equilibrium among several intestinal bacteria, as found when animals are fed a NFD, is accompanied by normalization of lipidaemic profile, lipoprotein re-arrangement and, finally, attenuated inflammation, which is associated with the altered lipidaemic profile.

The rationale of these responses is still to be better clarified. However, clear evidence exists that LPS affects the integrity of the intestinal mucosa by altering tight junctions and thereby impairing intestinal permeability [53,54,55,56]. In particular, evidence exists that alteration of gut microbiota induced by HFD leads to overproduction of LPS by Gram-negative bacteria, which is followed by impairment of the tight junction proteins, such as occludin, claudin-1 and ZO-1, which leads to LPS entering the portal circulation [57] and thereby producing at least two systemic responses: one is mediated by liver inflammation via TLR4 activation and cytokine release, which represent the key mechanisms of imbalanced packaging and release of lipoproteins from the liver [53,58,59]; the second one is represented by a condition of systemic inflammation and oxidative stress which leads to enhanced atherosclerosis and cardiometabolic risk [12,60,61].

These pathophysiological events are counteracted by bergamot extract and fibres. This is also consistent with previous data showing that BPE, a powerful antioxidant in vitro and in vivo, leads to significant protection of vasculature against oxidative damage subsequent to dyslipidaemia in both diet-induced metabolic syndrome and in patients undergoing increased cardiometabolic risk [48,62,63].

On the other hand, BPE has been found to produce relevant protection under conditions of liver inflammation, thereby preventing NASH and its deleterious effects in cardiometabolic risk, mostly due to its antioxidant properties [64].

Our data also show a synergistic response when prebiotic BMF is associated with BPE. Previous data have shown that BMF may produce a significant inhibition of post-prandial insulin response in patients and that this could account for a role of BMF in maintaining a normal metabolic balance in subjects suffering from metabolic syndrome [43].

On the other hand, the use of prebiotics is consistent with gut microbiota normalization able to reduce cardiometabolic risk [65,66,67,68].

Thus, it is likely that a combination of both antioxidant BPE and prebiotic BMF may better target inflammatory/oxidative damage and dysbiosis subsequent to HFD with a sequential response occurring via reduction in LPS release and subsequently by attenuating endotoxin-related systemic consequences.

In conclusion, our data confirm that HFD-related changes of gut microbiota are accompanied by increased body weight and alteration of lipoprotein size, an effect which is associated with modification of lipidaemic profile and imbalanced glucose levels. On the other hand, dysbiosis produced by alterations of gut microbiota and the subsequent alteration in lipidaemic profile and lipoprotein packaging contributes to the development of HFD-associated imbalance occurring in the mechanisms of lipid regulation and transport, occurring both at the intestinal and systemic level. This is also expressed by enhanced oxidative stress and increased LPS levels, thereby representing key mechanisms in systemic inflammation which may be found in animals fed HFD.

Our data also show, for the first time, that the alterations induced by HFD may be counteracted by supplementing rats with BPE, BMF or a combination of both, which restored gut microbiota and produced a re-arrangement of lipoprotein size, reduction in both LPS and MDA levels, and, finally, led to antagonism of diet-induced dyslipidaemia and metabolic imbalance. This suggests that combining antioxidants and prebiotics leads to sequential responses for better counteracting diet-induced alterations of gut microbiota and their deleterious effects on cardiometabolic risk profile.

## 4. Materials and Methods

### 4.1. Plant Material

Bergamot (botanical name: *Citrus bergamia* Risso & Poiteau, Variety Fantastico) is an endemic plant growing almost exclusively in the jonic cost of the Calabrian Region in the South of Italy. The bergamot polyphenolic-rich extract (BPE) and fibres (BMF) used throughout the study were obtained from fruits which were collected from December to January 2022 from plants located in a range of 90 Km from Bianco to Reggio Calabria, Italy (GPS coordinates: latitude: 38.0917; longitude: 16.15159).

In particular, bergamot juice (BJ) was obtained from peeled fruits by squeezing. The juice was oil-fraction-depleted through stripping and clarified through ultra-filtration, with subsequent loading on to a polystyrene resin column able to absorb polyphenol compounds of molecular weights between 300 and 600 Da (Mitsubishi Chemical, Cleansui Co., Ltd., Tokyo, Japan). Polyphenol fractions were eluted by a 1 mM KOH solution. The basic eluate was incubated on a rocking platform to reduce the furocoumarin amount. The phytocomplex was neutralized through filtration on cationic resin at acidic pH, vacuum dried and minced to the desired particle size to obtain BPE powder. The toxicological analyses performed, including heavy metal, pesticide, phthalate and synephrine content analyses, revealed the absence of toxic compounds at significant levels [40]. Standard microbiological tests showed that the final BPE and BMF were free of mycotoxins and contaminating bacteria. Moreover, studies for detecting acute and chronic toxicity in rodents for both products were conducted under (Good Laboratory Practice) GLP conditions. In particular, the study N.152-0041 was carried out in compliance with the EU Directive 2004/9/EC and Directive 2004/9/EC for GLP Guidelines and with OECD Guidelines for Repeated Dose 30-day Oral Toxicity Study in Rodents [69]. In addition, the study N.152-0043 was conducted with OECD Guidelines for Repeated Dose 90-day Oral Toxicity Study in Rodents [70]. Finally, micronuclei test, aberration test and reversed mutation measurements confirmed that both BPE and BMF were unable to produce toxicological or mutagenic effects in rodents according to current regulatory guidelines.

The antioxidant analysis of bergamot extract was performed prior to the administration of products in animals by means of EPR analysis (Supplementary S1, Figures S1 and S2).

Fibres obtained by bergamot albedo were micronized, and HPLC and nutritional analysis of the powder was performed. All materials were provided by H&AD srl (Herbal and Antioxidants Derivatives srl, Bianco, Italy). Test products were dissolved in water. A specification sheet of both extracts is shown in the Supplementary Materials (Supplementary S3 and S4).

### 4.2. High-Pressure Liquid Chromatography (HPLC)

HPLC analysis was performed by a Fast 1200 HPLC system (Agilent Technologies, 5301 Stevens Creek Blvd, Santa Clara, CA, USA) equipped with a DAD detector and a ZORBAX Eclipse XDB-C18 column—50 mm. A quantity of 2 µL of sample was injected and eluted with a two-solvent gradient of water and acetonitrile. Different gradients were used to determine flavonoid, furocoumarin and other polyphenol contents. The flow rate was 3 mL/min, and the column was maintained at 35 °C. The detector was monitored at 280 nm. Flavonoid and furocoumarin pure standards were purchased from Sigma-Aldrich (Burlington, MA, USA). Brutieridin and melitidin were identified according to Di Donna [40]. In the Supplementary Methods section, it is possible to view the chromatograms of HPLC analyses related to BPE and BMF powders. Method validation parameters, such as linearity, limits of detection, precision and accuracy, are available in Supplementary S2.

The main flavonoids identified were standardized at 48% in BPE powder (Supplementary S3). The estimated concentrations of the five main flavonoids were: neoeriocitrin (131,942 ppm), naringin (138,945 ppm), neohesperidin (134,617 ppm), melitidin (27,950 ppm) and brutieridin (56,496 ppm) (Supplementary S3).

The concentrations of the main flavonoids in BMF powder were standardized at 17%: neoeriocitrin (4398 ppm), naringin (5184 ppm), neohesperidin (3966 ppm), melitidin (1233 ppm) and brutieridin (2286 ppm) (Supplementary S4).

### 4.3. Animals

Male Sprague-Dawley 3-month-old rats (Charles River, Milan, Italy) weighing 270–290 g were used throughout the study. All animals were housed and cared for in accordance with the Italian National Health Ministry Guidelines on Laboratory Animal Welfare following the Italian regulations for the protection of animals used for experimental and other scientific purposes (D.L. 26/2014) as well as with the European Community guidelines [71]. The study was conducted according to the guidelines of the Declaration of Helsinki and approved by the local Ethics Committee (Calabrian Region Prot. 324: 12 October 2021) (Supplementary S5).

Rats were housed and maintained under controlled conditions of temperature (21 °C) and humidity (60–65%) with a 12 h light/12 h dark cycle and allowed food ad libitum.

The high-fat diet TD.88137 Total Fat (21% by weight; 42% kcal from fat) was purchased from Harlan Laboratories, Rossdorf, Germany; the BPE and BMF, both micronized and, when required, co-grinded, were kindly provided by H&AD srl (Herbal and Antioxidants Derivatives srl, Bianco, Italy).

### 4.4. Study Design

After adaptation, the rats were randomly allocated in eight experimental groups:

Control group, fed a normal-fat diet (NFD) for 4 consecutive weeks (n = 6);
HFD group, fed a high-fat diet (HFD) for 4 consecutive weeks (n = 6);
NFD receiving 20 mg/Kg of BPE for 4 consecutive weeks (n = 6);
NFD receiving 20 mg/Kg of BMF for 4 consecutive weeks (n = 6);
NFD receiving 20 mg/Kg of BPE + BMF for 4 consecutive weeks (n = 6);
HFD receiving 20 mg/Kg of BPE for 4 consecutive weeks (n = 6);
HFD receiving 20 mg/Kg of BMF for 4 consecutive weeks (n = 6);
HFD receiving 20 mg/Kg of BPE + BMF for 4 consecutive weeks (n = 6);

Quantities of 20 mg/Kg of the BPE powder were administered alone or in combination with 20 mg/Kg mg of power derived from dried albedo and pulp fibres (BMF).

All treatments were given via gastric gavage once daily over a period of four weeks. The doses of BPE and BMF used throughout the study and the treatment time were chosen according to previous data from our and other research groups [40,72,73].

Body weight, plasma glucose, total cholesterol, triglycerides, HDL, LDL, MDA, plasma lipoprotein size and concentrations were measured as previously shown [40,74] before starting treatment and at the end of the feeding period. Blood samples were collected by means of venipuncture of the tail vein and were stored as previously described [40].

At the same experimental times, rat faeces were collected from the anus of each rat using sterile EP tubes and immediately preserved in liquid nitrogen at basal and after 4 weeks of treatment in animals fed the NFD and HFD according to the treatment schedule.

### 4.5. Laboratory Measurements

At the baseline and after 4 weeks of treatment, a 12 h fasting morning blood sample was collected. All plasma marker concentrations or activities were measured using classical methods and commercial assay kits, according to the manufacturers’ instructions. Assay kits for total cholesterol, LDL-C, HDL-C, triglycerides, malondialdehyde (MDA), paraoxonase and glutathione peroxidase were purchased from Novamedical Srl (Reggio Calabria, Italy). All the laboratory tests were performed in a blinded manner in respect to the assigned treatment.

At the baseline and after 4 weeks of treatment with BPE, BMP and BPE + BMP, plasma samples were collected in EDTA tubes after a 12 h overnight fast: fasting plasma glucose (mg/dL), total cholesterol (mg/dL), low-density lipoprotein cholesterol (LDL cholesterol) (mg/dL), high-density lipoprotein cholesterol (HDL cholesterol) (mg/dL) and triglycerides (TGs) (mg/dL).

Plasma lipoprotein particles were detected as previously described [42] by means of the proton NMR spectroscopy technique with the simultaneous concentration measure of lipoprotein subclasses of different sizes. NMR provides calculated values for mean very-low-density lipoprotein (VLDL), LDL and HDL particle sizes plus estimates of total and VLDL, TGs and HDL cholesterol.

The oxidative stress index was assessed by plasma lipid peroxidation product malondialdehyde (MDA) through a lipid peroxidase assay kit (Sigma-Aldrich, Saint Louis, MO, USA) according to the manufacturer’s protocol. Briefly, the lipid peroxidation is determined through the reaction of MDA with thiobarbituric acid (TBA) to obtain a colorimetric (532 nm)/fluorometric (λex = 532/λem = 553 nm) product proportional to the MDA concentration in the sample [41].

LPS plasma levels at basal and after 4 weeks of treatment with BPE, BMF and BPE + BMF were detected through a competitive inhibition enzyme immunoassay. A Low Sample Volume Lipopolysaccharides (LPS) ELISA Kit (abbexa, abx355419, Leiden, NL) was used for the in vitro LPS quantification.

### 4.6. 16S rRNA Gene Sequencing and Analysis

#### 4.6.1. DNA Extraction and PCR Amplification

Rat faeces samples were processed using the E.Z.N.A.^®^ Stool DNA Kit (Omega Bio-tek, Norcross, GA, USA) to extract total microbial genomic DNA. Before being used, the DNA quality and concentration were assessed using 1.0% agarose gel electrophoresis analysis and a NanoDrop^®^ ND-2000 spectrophotometer (Thermo Scientific Inc., Waltham, MA, USA). An ABI GeneAmp^®^ 9700 PCR thermocycler (Thermo Scientific Inc., Waltham, MA, USA) was used to amplify the hypervariable region V3-V4 of the bacterial 16S rRNA gene using primer pairs 338F (5′-ACTCCTACGGGAGGCAGCAG-3′) and 806R (5′-GGACTACHVGGGTWTCTAAT-3′). Each sample was amplified in triplicate.

The PCR products were run on a 2% agarose gel and then purified through the GeneJET DNA Gel Extraction Kit (Thermo Fisher Scientific, Waltham, MA, USA). The quantification was performed using an AxyPrep DNA Gel Extraction Kit (Axygen Biosciences, Union City, CA, USA) according to the manufacturer’s instructions.

#### 4.6.2. Illumina MiSeq Sequencing

Once the amplicons were purified, they were combined in equimolar proportions and paired-end sequenced on the Illumina MiSeq PE300 platform (Illumina, San Diego, CA, USA) in accordance with standard protocols (Majorbio Bio-Pharm Technology Co. Ltd., Shanghai, China). The raw sequences were pre-analysed according to the BIPES protocol [75], and QIIME (1.8.0) was used to perform the following analyses. The representative sequences were aligned through PyNAST algorithms [76]. Phylogenetic relationships were assessed by FastTree sequence alignment [77], and the taxonomic assignments were combined to construct the BIOM file [78].

### 4.7. Statistical Analysis

Data analyses were performed using GraphPad PRISM 9.3.1 (GraphPad Software, Inc., La Jolla, CA, USA). The differences among treatments were evaluated using GraphPad PRISM 9.3.1 (GraphPad Software, Inc., La Jolla, CA, USA). The Shapiro–Wilk test was used to test normality. Normally distributed data were analyzed by one-way ANOVA followed by Tukey’s test; data not normally distributed were analyzed through the Kruskal–Wallis test followed by Dunn’s tests. The results are shown as means ± SEs. A *p*-value < 0.05 was considered statistically significant.

## Data Availability

The data presented in this study are available upon request from the corresponding author.

## Supplementary Materials

The following supporting information can be downloaded at: https://www.mdpi.com/article/10.3390/ijms241612967/s1.
