# Supplementary material for: Bergamot Polyphenolic Extract Combined with Albedo and Pulp Fibres Counteracts Changes in Gut Microbiota Associated with High-Fat Diet: Implications for Lipoprotein Size Re-Arrangement
Source: Int J Mol Sci. 2023 Aug 19;24(16):12967. doi: 10.3390/ijms241612967 (PMC10454550; doi:10.3390/ijms241612967)
Supplement: Supplementary file 1 [file ijms-24-12967-s001.zip › Supplementary S1.pdf]

Materials and methods

The antioxidant capability of the Bergamot polyphenolic extract (BPE) has been assessed through Electron Paramagnetic Resonance (EPR), a spectroscopy technique which allows to identify and quantify radical species. Notably the scavenging activity was evaluated against DPPH and hydroxyl ($^{\bullet}OH$) radicals, as previously described (https://doi.org/10.3390/plants12010027).

DPPH radical scavenging activity of BPE was assessed adding 50 µL of fraction to 200 µL of methanolic DPPH solution (1 mM). Ascorbic acid, due to its high well known antioxidant power, has been used as positive control.

Moreover, the capability of BPE to neutralize hydroxyl radical ($^{\bullet}OH$) was evaluated. Hydroxyl radicals were generated by the Fenton reaction, and BMPO (5-tert-Butoxycarbonyl-5-methyl-1-pyrroline-N-oxide, B568-10, Dojindo EU GmbG, Munich, Germany) was used as a spin trap.

The acquisitions were carried out after mixing the BMPO solution (15 µL), H_2O_2 1mM (75 µL), Iron (II) sulfate heptahydrate 100 µM (75 µL) and ddH_2O (50 µL). 50 µL of BPE were added to reaction mixture after the production of the hydroxyl radical. Ascorbic acid (5mg/mL) was used as positive control.

EPR spectra were acquired using a Bruker Magnettech ESR5000 (Bruker Biospin MRI GmbH, Ettlingen, Germany) with the following experimental parameters: 9.43 GHz X-band, 0.05 mT modulation amplitude, 336.64 mT central field, 12.00 mT sweep, 30 s sweep time, modulation frequency of 100 Khz, 20 mW microwave power (for DPPH radical) or 6 mW microwave power (for BMPO-OH).

The integration of EPR spectral area was performed using OriginPro 2018 (OriginLab Corporation, Northampton, MA, USA) to evaluate the amount of free radicals in each acquisition.

Results

In DPPH-EPR assay, a six-line pattern DPPH spectrum was obtained with and integrated spectral area value of 1184.92 a.u. (Figure 1). BPE (5 mg/mL) reduced the DPPH signal intensity (30.53 a.u., Figure 1), as well as Ascorbic acid (32.13 a.u., Figure 1), that it is well known for its high antioxidant power.

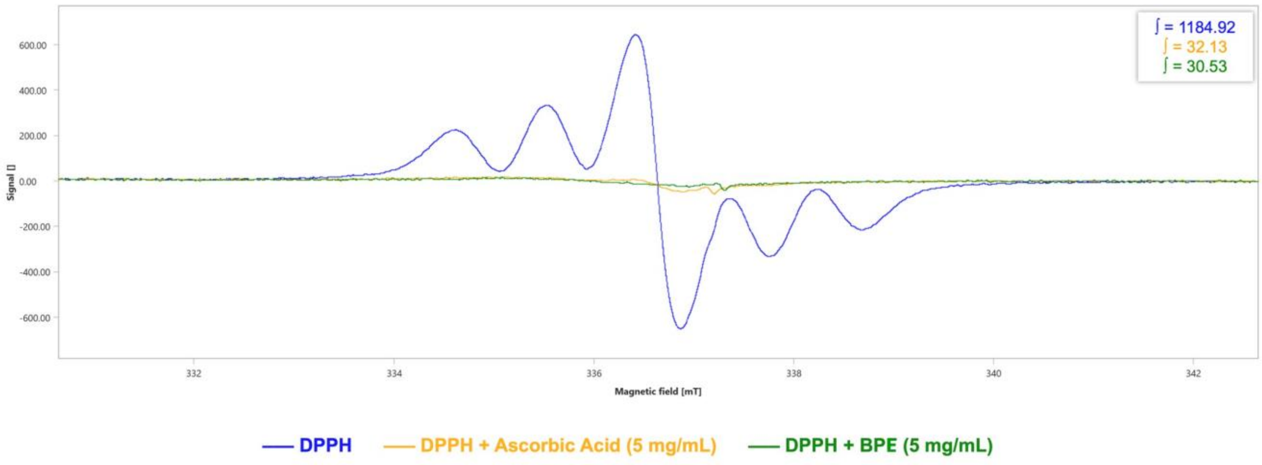

Figure S1. DPPH-EPR assay. EPR spectra and respective spectral areas for DPPH in absence (blue) and presence of BPE (green) or ascorbic acid as positive control (yellow)

Furthermore, we assessed the scavenging activity against hydroxyl radical (OH). In this latter assay the typical 4-line spectrum of the BMPO-OH was obtained and the integration of spectral area showed a value of 42.32 a.u. (Figure 2A). The addition of BPE (5 mg/mL) reduced the hydroxyl radical concentration, as confirmed by the decreased spectral area (23.73 a.u., Figure 2B). Likewise to the DPPH assay, the BPE scavenging activity against hydroxyl radical is similar to those exerted by Ascorbic acid (22.17 a.u., Figure 2C).

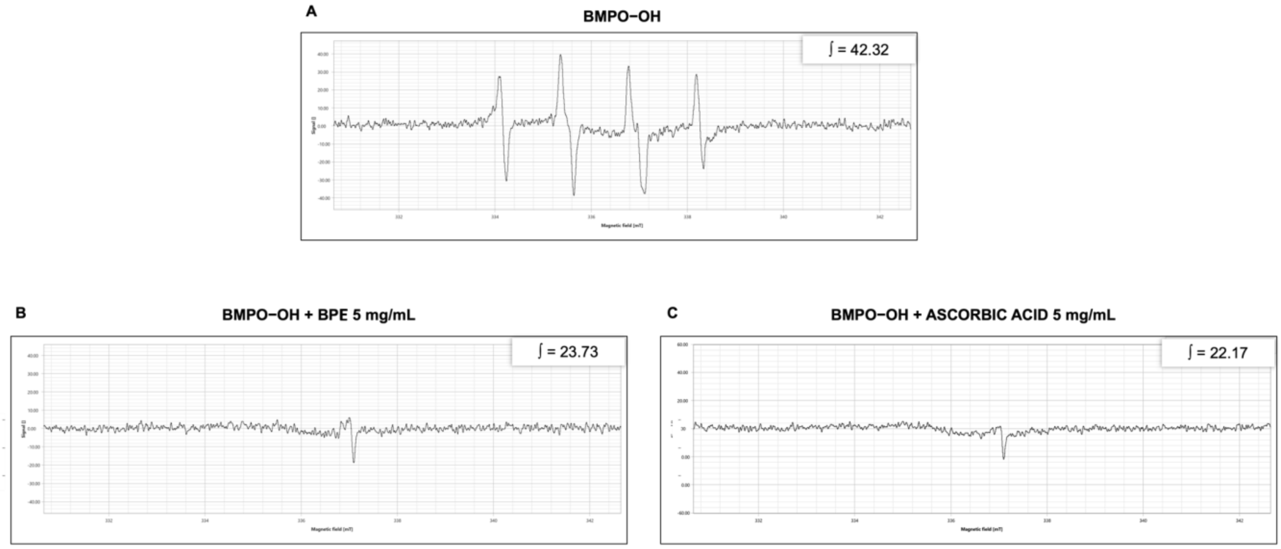

Figure S2. EPR spin trapping assay for scavenging activity against hydroxyl radical (OH). EPR spectra and respective spectral areas for BMPO-OH adduct **(A)**, BMPO-OH + BPE **(B)** and BMPO-OH + Ascorbic Acid **(C)**.
