# Supplementary material for: Bergamot Polyphenolic Extract Combined with Albedo and Pulp Fibres Counteracts Changes in Gut Microbiota Associated with High-Fat Diet: Implications for Lipoprotein Size Re-Arrangement
Source: Int J Mol Sci. 2023 Aug 19;24(16):12967. doi: 10.3390/ijms241612967 (PMC10454550; doi:10.3390/ijms241612967)
Supplement: Supplementary file 1 [file ijms-24-12967-s001.zip › Supplementary S3.pdf]

# SPECIFICATION SHEET

| | | |
|---|---|---|
| **ID Code** | **BRGFBRDC** | |
| **Trade name** | **BERGAMOT FIBER** | |
| **DESCRIPTION** | **SPECIFICATIONS** | |
| Botanical Source | Citrus Bergamia Risso et Poit. | |
| Family | Rutaceae | |
| Country of Origin | Calabria, Italy | |
| Part Used | Fruit | |
| Shelf Life | 2 years, if correctly stored | |
| **ORGANOLEPTIC CHARACTERISTICS** | **SPECIFICATIONS** | |
| Colour | Yellow Powder | visual (CQ-MO-148) |
| Odour | Aromatic | visual (CQ-MO-148) |
| Flavour | Characteristic of bergamot | sensory (CQ-MO-148) |
| **CHEMICAL CHARACTERISTICS** | **SPECIFICATIONS** | **METHOD** |
| Appearance | Powder | |
| Mesh Size | Pass 50 mesh | Sieve: (CQ-MO-023) |
| Tapped Density | 50 - 70 g/100mL | PT CHIM 65 rev 0 2011 |
| Moisture Content | < 10.0% | ISTISAN 96/34, pag 7 |
| Organic Solvent Residue | Complies with Dir. 2009/32/CE | GC: (CQ-MO-168) |
| Pesticides Residue | Complies with Reg. UE 839/2008 | PT CHIM 69rev 02 011 |
| Pectin | > 15.0% | IM |
| Tot. active ingredient strength (Neoeriocitrin, Naringin, Neohesperedin, Melitidin, Brutieridin) | > 1.5 % | HPLC |
| **HEAVY METALS** | **UNIT** | **RANGE** |
| Arsenic | mg/kg | <1.0 |
| Lead | mg/kg | <0.5 |
| Cadmium | mg/kg | <0.5 |
| Mercury | mg/kg | <0.3 |
| **MICROBIOLOGICAL EVALUATION** | | |
| Aerobic Plate Count | <10,000 CFU/g | ISO 4833-1:2013 |
| Yeast and Mold Count | <1,000 CFU/g | ISO 21527-1:2008 |
| E. Coli | Negative | ISO 16694-2:2001 |
| Coliform | Negative | ISO 4832:2006 |
| Salmonella | Negative | UNI EN ISO 6579:2000 |
| Staphylococcus Aureus | Negative | UNI EN ISO 6888-2:2004 |
| Streptococci | Negative | PT BAT26 rev0 02012 |
| **PRODUCT TREATMENT** | | |
| Drying Method | **Hot air** | |

# *NUTRITIONAL FACTS*

| | |
|---|---|
| **PRODUCT NAME:** | **Bergamot Fiber** |
| **IDENTIFICATION CODE:** | **BRGFBRDC** |
| **NUTRIENTS** | **AMOUNT per 100g** |
| Calorific Value (kcal) | **150 - 400** |
| Fat (%) | **< 3.0** |
| Ash (%) | **< 7.0** |
| Total Carbohydrates (%) | **> 40.0** |
| Protein (%) | **< 10.0** |
| Water (%) | **< 10.0** |

RECOMMENDED STORAGE CONDITIONS:

Store in a fresh and dry place, repaired from light and humidity sources.

BEST BEFORE:

24 months under the previously mentioned conditions and in its original packaging.

PACKAGING:

15 kg bags.

Giuseppe Trunfio
H&ad srl analytical chemistry & processes
**E-mail : g.trunfio@head-sa.com**

# SAMPLE REPORT

| | |
|---|---|
| Acquisition Date/Time | 14-Oct-22 09:27:10 |
| Acquisition Method | Flavonoids ca. on Naringin_March22 |
| Chromera Version | 4.2.0.6415 |
| Dilution Factor | 21 |
| Report Date/Time | 19-Oct-22 10:59:44 |
| Sample Name | Bergamot Fiber Lot. 1402-22 |
| Vial Number | 53 |

Bergamot Fiber Lot. 1402-22 : 284:10:400:10 : 1

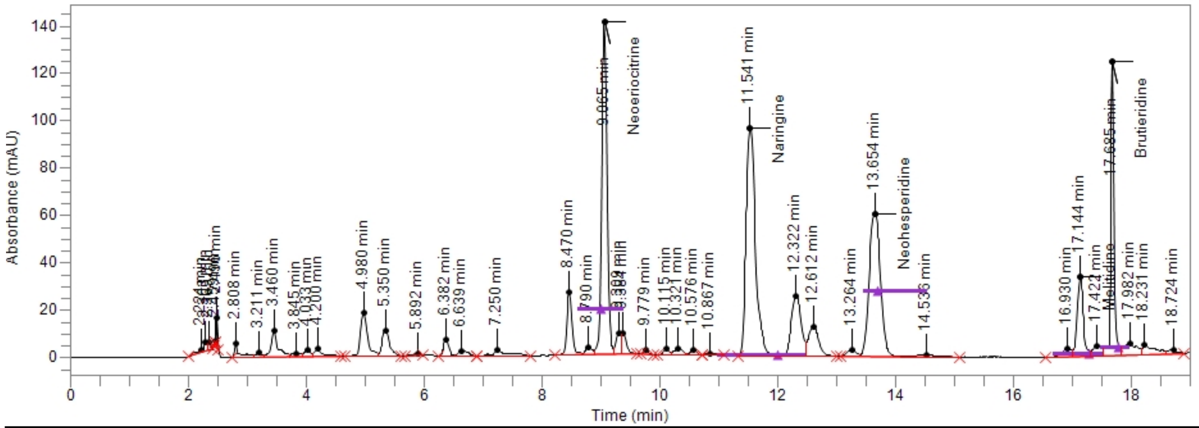


| Peak # | Time | Component Name | Area | Height | Final Amount | Units |
|---|---|---|---|---|---|---|
| 37 | 17.685 | Brutieridine | 460,428.7 | 124,257.2 | 2,286.9272 | ppm |
| 35 | 17.144 | Melitidine | 240,632.0 | 33,864.1 | 1,233.3295 | ppm |
| 28 | 11.541 | Naringine | 1,064,965.6 | 96,201.0 | 5,184.8012 | ppm |
| 20 | 9.065 | Neoeriocitrine | 900,833.9 | 140,543.2 | 4,398.0290 | ppm |
| 32 | 13.654 | Neohesperidine | 810,831.1 | 60,168.6 | 3,966.5970 | ppm |
| **Total** | | | 3,477,691.3 | | 17,069.6840 | |

# SAMPLE REPORT

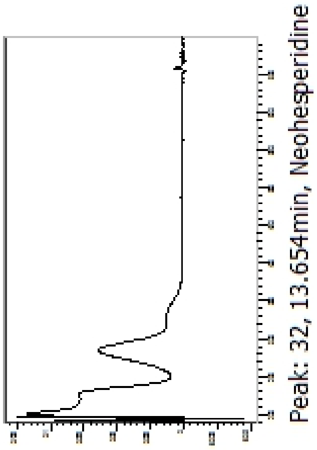


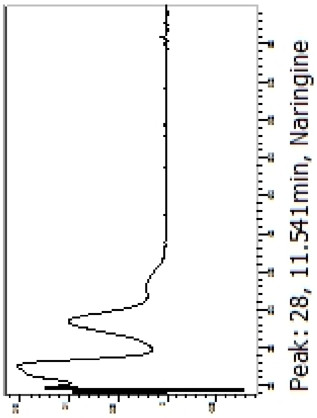


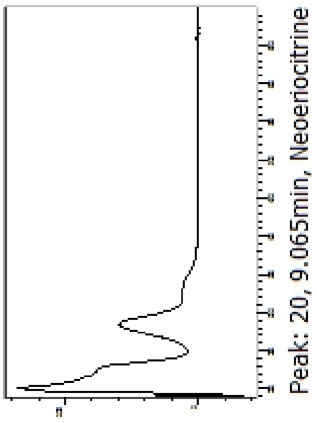


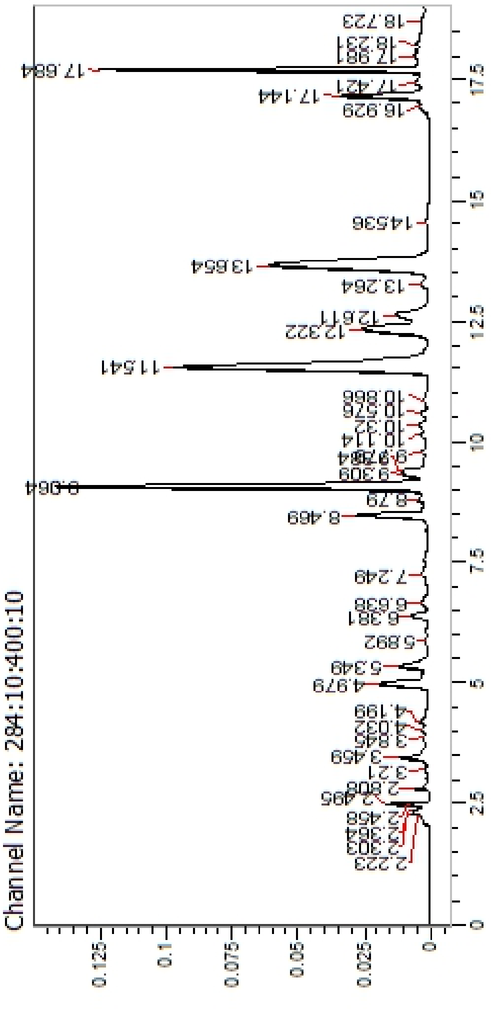


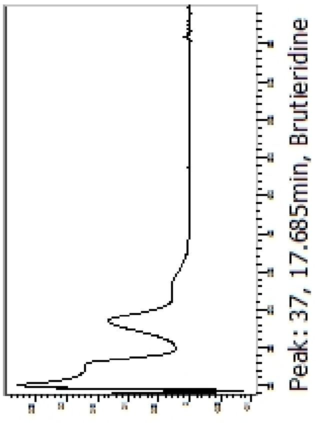


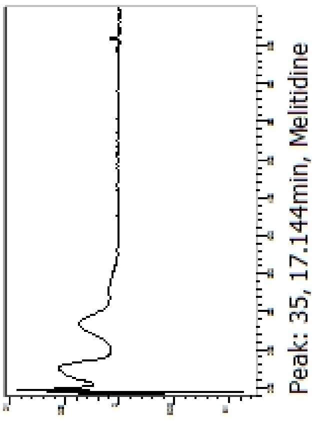

# SAMPLE REPORT

Approved:

Signature: ________________ Name: ________________ Date: _____________
