# Supplementary material for: Bergamot Polyphenolic Extract Combined with Albedo and Pulp Fibres Counteracts Changes in Gut Microbiota Associated with High-Fat Diet: Implications for Lipoprotein Size Re-Arrangement
Source: Int J Mol Sci. 2023 Aug 19;24(16):12967. doi: 10.3390/ijms241612967 (PMC10454550; doi:10.3390/ijms241612967)
Supplement: Supplementary file 1 [file ijms-24-12967-s001.zip › Supplementary S4.pdf]

Gradient hplc method

S. No.	Step	time (min)	Flow (mL/min)	Eluent A (%)	Eluent B (%)
0	Equilibration	7	1.1	15	85
1	Run	4	1.1	22	78
2	Run	8	1.1	22	78
3	Run	4	1.1	60	40
4	Run	1	1.1	100	0
5	Run	1	1.1	100	0
6	Run	1	1.1	10	90
7	Washing	1	1.1	10	90

Eluent A: Acetonitrile

Eluent B: 0.88% Trifluoroacetic acid

Linear regression: y = 2726X - 2256

Slope	Y interc.
2726	2256

	Naringin content in BPE sample				
	Replicate 1	Replicate 2	Replicate 3	Replicate 4	Replicate 5
Area	1333599,6	1312074,7	1313010,8	1313180,7	1311907,6
Conc. (mg/L)	488,3872	480,4911	480,8345	480,8968	480,4298

		Naringin content in test for accuracy		
			Replicate 1	Replicate 2
% of target	95,6%	Area	1278226,5	1265481,4
Conc. (mg/L)	461,2827	Conc. (mg/L)	468,0743	463,3989

		Naringin content in test for accuracy		
			Replicate 1	Replicate 2
% of target	116,3%	Area	1496949,1	1498968,1
Conc. (mg/L)	561,2827	Conc. (mg/L)	548,3100	549,0507

	Average Area	Standard Deviation	RSD (%)
Replicate 6			
1322601,6			
484,3528	482,5654	3,22	0,67%

	Average Area	Standard Deviation	RSD (%)	Recovery (%)
Replicate 3				
1267612,9				
464,1808	465,2180	2,50	0,54%	100,85%

	Average Area	Standard Deviation	RSD (%)	Recovery (%)
Replicate 3				
1495493,9				
547,7762	548,3790	0,64	0,12%	97,70%
