# Supplementary material for: Bergamot Polyphenolic Extract Combined with Albedo and Pulp Fibres Counteracts Changes in Gut Microbiota Associated with High-Fat Diet: Implications for Lipoprotein Size Re-Arrangement
Source: Int J Mol Sci. 2023 Aug 19;24(16):12967. doi: 10.3390/ijms241612967 (PMC10454550; doi:10.3390/ijms241612967)
Supplement: Supplementary file 1 [file ijms-24-12967-s001.zip › Supplementary S5.pdf]

REGIONE CALABRIA
COMITATO ETICO SEZIONE AREA CENTRO
e-mail: michelangelo.rossano@regcal.it---mancgino@libero.it

REGIONE CALABRIA

REGIONE CALABRIA

Registro Protocollo n. 324 del 12 Ottobre 2021

Oggetto: Verbale: ***Pre-clinical and cCinical Study on the effect of Bergamot Polyphenolic Fraction (BPF) and/or Bergamot Fibres (BMF) on gut microbioma and lipid metabolism***

Principal investigator: Prof. Vincenzo Mollace

Documenti Esaminati:

- Lettera d'Intenti;
- Protocollo;
- Sinossi;
- Quotazione studio Bergacyn;
- Lettera al Medico curante;
- Dichiarazione no Profit;
- Cv Prof.Pujia;
- Consenso trattamento dati,
- Conflitto di interessi.

Verificato:
il numero legale (come da elenco allegato) e che i Componenti del C.E. per i quali sussiste un conflitto di interesse di tipo diretto e indiretto si astengano nel pronunciare un parere.

Il giorno 24.09.2018 il Comitato Etico Regione Calabria Sezione Area Centro con sede presso l'A.O.U. Mater Domini in Via Tommaso Campanella, 115 Catanzaro, sulla base della relazione svolta e verificata la documentazione inviata dal Promotore, **esprime parere favorevole allo Studio Pre-clinico e Clinico di cui all'oggetto.**

Il C.E. è un organismo indipendente, senza rapporti di subordinazione gerarchica nei confronti della struttura in cui opera.

Esso effettua la valutazione degli aspetti etici della ricerca Biologica e clinica, nel rispetto del Decreto Legislativo n. 211 del 2003, della dichiarazione di Helsinki nella sua versione più aggiornata, della convenzione di Oviedo, delle norme di buona pratica clinica di cui all'allegato 1 al D.M. 15 luglio 1997, delle linee guida aggiornate dall'Agenzia europea per la valutazione dei medicinali in tema di valutazione dell'efficacia delle sperimentazioni cliniche e delle raccomandazioni del Comitato Nazionale di bioetica, formulando raccomandazioni, suggerimenti e pareri (questi ultimi in presenza di formale richiesta) su fondamentali e specifiche problematiche di natura etica.

L'organizzazione ed il funzionamento sono quelli previsti dal D.M. dell'08 Febbraio 2013

Si trasmette la presente per gli adempimenti del caso, secondo le rispettive competenze.

Resp. Segreteria Tecnico-Scientifica
Comitato Etico
(Dott. Luigi Mancuso)

Il Presidente
Comitato Etico
(Prof. Pietrantonio Ricci)
